# Genome-wide expression profiling of maize in response to individual and combined water and nitrogen stresses

**DOI:** 10.1186/1471-2164-14-3

**Published:** 2013-01-16

**Authors:** Sabrina Humbert, Sanjeena Subedi, Jonathan Cohn, Bin Zeng, Yong-Mei Bi, Xi Chen, Tong Zhu, Paul D McNicholas, Steven J Rothstein

**Affiliations:** 1 Department of Molecular and Cellular Biology, University of Guelph, Guelph, Ontario, N1G 1K4, Canada; 2 Department of Mathematics and Statistics, University of Guelph, Guelph, Ontario, N1G 1K4, Canada; 3 Syngenta Biotechnology Inc, 3054 Cornwallis Rd, Research Triangle Park, NC, 27709, USA; 4 Current Address: Pioneer Hi-Bred, 7000 62nd Ave, Johnston, Iowa, 50131, USA

**Keywords:** Drought, Maize, Nitrogen, Microarray, Clustering analysis, Abiotic stress

## Abstract

**Background:**

Water and nitrogen are two of the most critical inputs required to achieve the high yield potential of modern corn varieties. Under most agricultural settings however they are often scarce and costly. Fortunately, tremendous progress has been made in the past decades in terms of modeling to assist growers in the decision making process and many tools are now available to achieve more sustainable practices both environmentally and economically. Nevertheless large gaps remain between our empirical knowledge of the physiological changes observed in the field in response to nitrogen and water stresses, and our limited understanding of the molecular processes leading to those changes.

**Results:**

This work examines in particular the impact of simultaneous stresses on the transcriptome. In a greenhouse setting, corn plants were grown under tightly controlled nitrogen and water conditions, allowing sampling of various tissues and stress combinations. A microarray profiling experiment was performed using this material and showed that the concomitant presence of nitrogen and water limitation affects gene expression to an extent much larger than anticipated. A clustering analysis also revealed how the interaction between the two stresses shapes the patterns of gene expression over various levels of water stresses and recovery.

**Conclusions:**

Overall, this study suggests that the molecular signature of a specific combination of stresses on the transcriptome might be as unique as the impact of individual stresses, and hence underlines the difficulty to extrapolate conclusions obtained from the study of individual stress responses to more complex settings.

## Background

Last year, the world population reached 7 billion and it is expected to exceed 10 billion by 2100 [1]. Food supply is critical if we are to sustain this population. Twentieth century agriculture met the growing food demand by achieving sustained increases in crop yields. In the past two decades however, the difficulty in maintaining the required yield increases along with the high environmental and economical price of intensive production methods have become strong incentives for a profound change. The challenge lies not only in increasing production but also in developing economically and environmentally sustainable practices.

Agriculture is by far the biggest user of water, and accounts for almost 70 percent of all withdrawals worldwide. According to the International Panel on Climate Change, water availability will be problematic in the years to come as the timing and geographical patterns of precipitations are predicted to undergo significant changes, along with the increased frequency of droughts, the amount of snowmelt runoff and temperature-induced evaporation [2]. Access to irrigation water will be one of the main challenges of this century and its use will have to be carefully managed to maintain crop production in drought-prone areas.

The use of fertilizer in agriculture has increased fivefold since the 1960s and about 65 percent of it is used on cereals. Nitrogen plays a major role in plant nutrition but it is the mineral element most often deficient in arable soils. Although it is directly linked to cereal crop yield, inadequate or inefficient fertilization is a major contributor to soil degradation and represents substantial financial losses worldwide. Further, the very significant loss of added nitrogen fertilizer to the air and water causes very significant global environmental damage. Therefore efficient use is required both to enhance yields as well as to limit the environmental damage caused by crop production.

Corn (*Zea mays L*.) is the most important crop in terms of area planted (almost 160 million ha in 2009) and production (820 tonnes in 2009) before rice and wheat. Although it is highly demanding in water and fertilizer, it is widely grown in areas where environmental conditions are far from ideal, and productivity therefore depends on large amounts of inputs. While in some places excessive irrigation and fertilization are clearly common practice in order to guarantee yield, in other regions large gaps remain between achievable and realized yield. In both cases, inadequate use of inputs comes at high environmental and economical costs and a better management of available resources is needed to maximize profits and meet the challenges of modern agriculture.

To implement better agricultural practices, numerous models have been built that help predict productivity while optimizing the timing and quantity of inputs required (for example [3,4]). Their empirical formulas integrate various parameters such as water availability, carbon and nitrogen pools, as well as the dynamics between them, based on the analysis of large experimental datasets obtained in the field. Studies focusing on the effects of irrigation in combination with nitrogen fertilizer application for example have been instrumental in designing those models. They have shown in particular that the dynamic interaction between the two factors clearly impacts plant physiology and leads to a multiplicative increase in grain yield and water use efficiency (for example, [5]). Yet very little is known about the underlying molecular mechanisms that contribute to those increases in response to the availability of water and nitrogen. With the development of genomic resources, numerous transcript profiling experiments have been performed on plant response to nutrient or water deficiency. Lists of genes responsive to nitrogen have been generated in *Arabidopsis*[6-11], rice [12,13] and other species (see [14] for review). Similarly, the effects of drought have been surveyed in *Arabidopsis*[15-17] rice [18,19], and corn [20,21]. The few studies that examined the potential interactive effects of abiotic stresses, which are usually present simultaneously during the field season, mostly focused on the combination of heat and water stresses (for example, [22,23]) and allowed the making of significant progress in that area. To our knowledge, the present study is unique in exploring the genome-wide expression profile of corn plants subjected to individual and combined nitrogen and water stresses in a controlled environment. The information generated is relevant in the context of crop improvement and should provide valuable insight more specifically for current research and biotech approaches.

## Results

### Experimental Design

Plants were grown in the greenhouse using a setup that aimed at controlling the amount of both nutrient and water while trying to best mimic natural conditions. To achieve proper development of the root system while being able to supply precise amounts of fertilizer and water, a clay-based substrate (Turface) was chosen instead of a complete hydroponic system. Stress and recovery conditions were empirically determined as explained below.

Optimal and limiting nitrogen conditions were defined respectively as 20 mM and 8 mM NH_4_NO_3_ and applied at the beginning of the experiment as indicated in the Methods. The limiting nitrogen condition caused a chronic, moderate stress that translated into a phenotype visible around week 4. Nitrogen-deprived plants were smaller and lighter green compared to control plants (Additional file 1), but since the stress applied was only moderate, these plants were able to develop normally and go through a complete life cycle as would be expected in a field setting.

Water conditions were also defined empirically so as to achieve visible but potentially reversible physiological changes. The chosen conditions were not lethal and allowed complete recovery once water was supplied again, as might typically be the case several times over the course of a growing season. Two drought levels were achieved by withdrawing water for 3 d (mild stress) and 5 d (severe stress) before sampling. Leaf rolling and wilting were clearly observable on severely stressed plants, while moderately stressed plants only displayed intermediate effects (Additional file 1). Two re-watering time points were tested to assess plant recovery from drought stress. Severely stressed plants were watered again 2 h and 5 h before sampling. After 2 h rehydration, plants could barely be visually distinguished from the well-watered controls while after 5 h rehydration they could not be distinguished at all from the controls.

**Figure 1:**
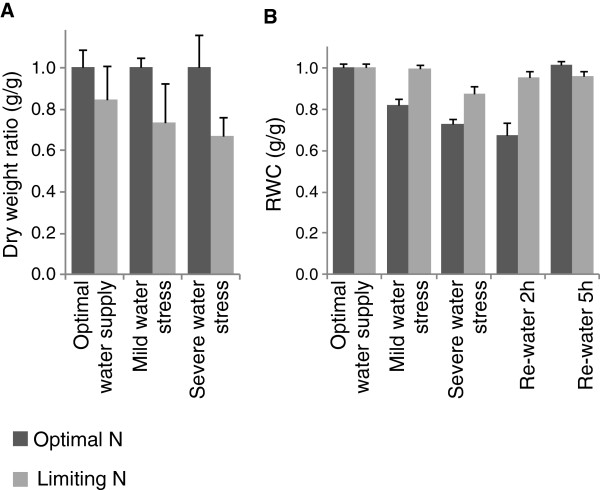
**Physiological measurements. A**. Plant dry weight under different water regimes relative to the average value of the corresponding optimal nitrogen treatment. Bars represent the standard error of 3 biological replicates. **B**. Relative Water Content in the first fully developed leaf under different water regimes relative to the average value of the corresponding optimal water treatment. Bars represent the standard error of 4 biological replicates.

To assess the physiological state of stressed plants compared to untreated controls, a series of measurements were performed. Dry weight loss was quantified as a measure of chronic nitrogen limitation. The data in Figure 1-A show the ratio of dry weights measurements relative to the corresponding optimal nitrogen averages. The limiting nitrogen treatment resulted in 15 to 30% loss in dry weight. The loss was more pronounced under water stress conditions than under optimal water supply although the difference was not found to be statistically significant.

The impact of water limitation was estimated by measuring the relative water content (RWC) in the first fully expanded leaf. Under our experimental conditions, the RWC under optimal water supply were 96.1% (+/− 2.0%) and 93.6% (+/− 2.0%) when plants were grown respectively at optimal and limiting nitrogen concentrations. In Figure 1-B, the results are expressed as ratios of the calculated RWC in each condition relative to the corresponding value under optimal water regime. Under optimal nitrogen conditions, RWC was found to decrease markedly in response to water stress, respectively about 15% drop under mild and 30% under severe stresses. The decrease was less drastic when plants were grown under limiting nitrogen conditions but still significant under severe water stress (about 15% reduction in RWC compared to the corresponding optimal water treatment). When plants were watered again, the RWC was restored gradually to non-stress levels. This trend was in agreement with the phenotypes described previously upon re-watering.

Applied independently, the chosen nitrogen and water limitation conditions allowed visible physiological changes and led to measurable effects on dry weight and RWC, which were in the same range of about 15 to 30% loss relative to the optimal.

### Microarray Analysis

A transcript profiling experiment was performed using a customized Affymetrix maize microarray containing 46784 entities. About 70% of all entities on the chip were associated with functional annotation at the time of the analysis (see Methods for a more detailed description of this experiment). Thirty conditions were studied, each being a unique combination of three parameters: organ (leaf, root or stem), nitrogen supply (optimal or limiting) and water supply (optimal, mildly stressed, severely stressed, severely stressed followed by 2 h re-watering, or severely stressed followed by 5 h re-watering). Each condition was run in a set of three biological replicates, totalling 90 samples. Raw data files (.cel files) were imported in Genespring GX (Agilent, CA, USA). Eighty-eight out of the 90 samples were exploitable and used in the analysis, so that each unique combination of conditions was represented by at least 2 replicates. The data were normalized using RMA (Robust Multichip Average) and log-transformed. Baseline transformation of the normalized signal was then performed to the median of all samples. Additional file 2 shows the Box Whisker plot of the normalized intensities for all chips. Pre-filtering was performed on the raw data to filter out probes that were consistently detected at low levels, and an arbitrary detection threshold of 20 signal intensity unit was used. Out of 46784 entities, 39664 had a raw signal above 20 in 100% of the replicate values in at least one out of the 30 conditions. This list was used in all subsequent analyses. For homogeneity, differentially expressed genes were identified by first filtering on a fold change ≥ 2 and then performing statistical tests using the Benjamini-Hochberg multiple testing method with a false discovery rate set at 0.05.

### Identification of differentially regulated entities in response to nitrogen limitation and different levels of water stresses and recovery

In this experiment, plant transcriptional responses to stress were examined in the leaf, stem and root. The different roles of these three organs in the plant as well as the transcriptional trends observed suggested very distinct responses to stress at the molecular level and we therefore performed separate analyses for each organ. The response to nitrogen and water stresses were first analysed independently, keeping the other parameter optimal.

The impact of nitrogen limitation on the transcriptome of each organ was studied using the six treatment combinations where plants did not undergo any water stress (*i.e.* optimal and limiting nitrogen supply in leaf, root and stem). For each organ, genes that displayed a fold change ≥ 2 when comparing optimal vs. limiting nitrogen and a t-test p-value corrected for multiple testing below 0.05 were retained (see Methods for a detailed description of the procedure). As shown in Table 1-A, very few genes passed these criteria under our conditions: only 68, 17 and 6 entities respectively were found to be responsive to chronic nitrogen limitation in leaf, root and stem.

**Table 1:** Impact of nitrogen and water stresses on leaf, root and stem transcriptomes

**A**			
	Nitrogen limitation		
Leaf	68		
Root	17		
Stem	6		
Total	90		
	(0.2%)		
**B**			
	Mild Water Stress	Severe Water stress	Total
Leaf	5151	4954	6841
			(15.8%)
Root	2370	5994	6262
			(12.8%)
Stem	1539	8767	8903
			(19.0%)
Total	7401	13927	
	(15.8%)	(29.8%)	

Number of entities differentially regulated under limiting nitrogen (water being optimal) (A) and drought stress (nitrogen being optimal) (B). Numbers in parentheses indicate percentages relative to the total number of entities on the chip.

The impact of drought was then assessed using 9 of the 30 conditions, where nitrogen supply was optimal and dismissing re-watered samples from the analysis (*i.e.* optimal water supply, mild and severe water stresses in leaf, root and stem). For each organ, a similar procedure as described above was followed. Respectively 6841, 6262 and 8903 entities were found to be differentially regulated under mild or severe water stress in leaf, root and stem (Table 1-B).

While both nitrogen and water stresses induced observable phenotypical effects of similar magnitude compared to their optimal, our data show that the impact of those treatments on the transcriptome was extremely different (Table 1). The number of genes differentially expressed under nitrogen limitation was very low (0.2% of all chip entities) whereas the various water stress treatments widely affected gene expression. Overall, 15.8% and 29.8% of chip entities respectively were found to vary significantly from the well-watered control under mild and severe water stresses. This result was most likely due to our particular experimental setup. In an attempt to mimic field conditions, nitrogen stress was applied in a mild and chronic fashion while water stress was much more sudden. Under nitrogen stress, transcriptional changes are thus expected to be limited at the time of sampling since the plant has had a considerable time to adjust to stress, which is obviously not the case under water stress.

**Figure 2:**
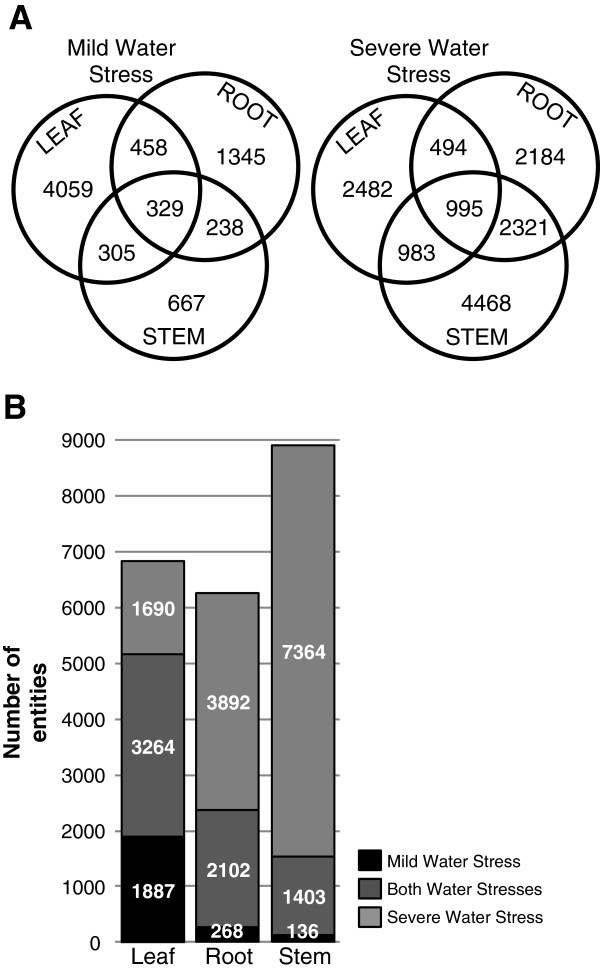
**Impact of water stresses on leaf, root and stem. A**. Venn diagrams showing the overlap of the response to water stresses between organs. **B**. Graph showing the response to mild and severe water stresses in each organ. Numbers correspond to entities differentially expressed under the indicated conditions.

Each tissue displayed a distinct behaviour at the transcriptome level in response to drought. Quantitatively, the leaf transcriptome was the most strongly affected by a mild water stress (5151 entities differentially expressed) but quite surprisingly this response was not aggravated by a severe stress (4954 entities) (Table 1-B and Figure 2-B). This was the case however for root and stem, where the number of differentially expressed entities increased with the acuteness of the stress (2370 to 5994 entities for root, and 1539 to 8767 entities for stem).

The extent of the induced genes overlap between different organs was found to be rather limited relative to the total number of induced entities (Figure 2-A) and therefore also supported the idea that the response to drought is unique to each organ. Under mild water stress, most differentially regulated genes were unique to leaf (4059 entities, Figure 2-A). Under severe stress however, most differentially regulated genes were identified solely in stem (4468 entities) and the overlap of regulated genes was highest between stem and root (2321 entities in common, Figure 2-A). In addition, Figure 2-B shows that in leaf, most entities were common between mild and severe stresses, whereas in root and stem most entities were only identified under severe stress. This would suggest that the transcriptional response of the plant to water stress might have been first initiated in leaf, which already expressed most transcriptional changes under milder stress conditions, while in root and stem, gene regulation was triggered under more acute stress conditions and to a larger extent than in leaf.

**Figure 3:**
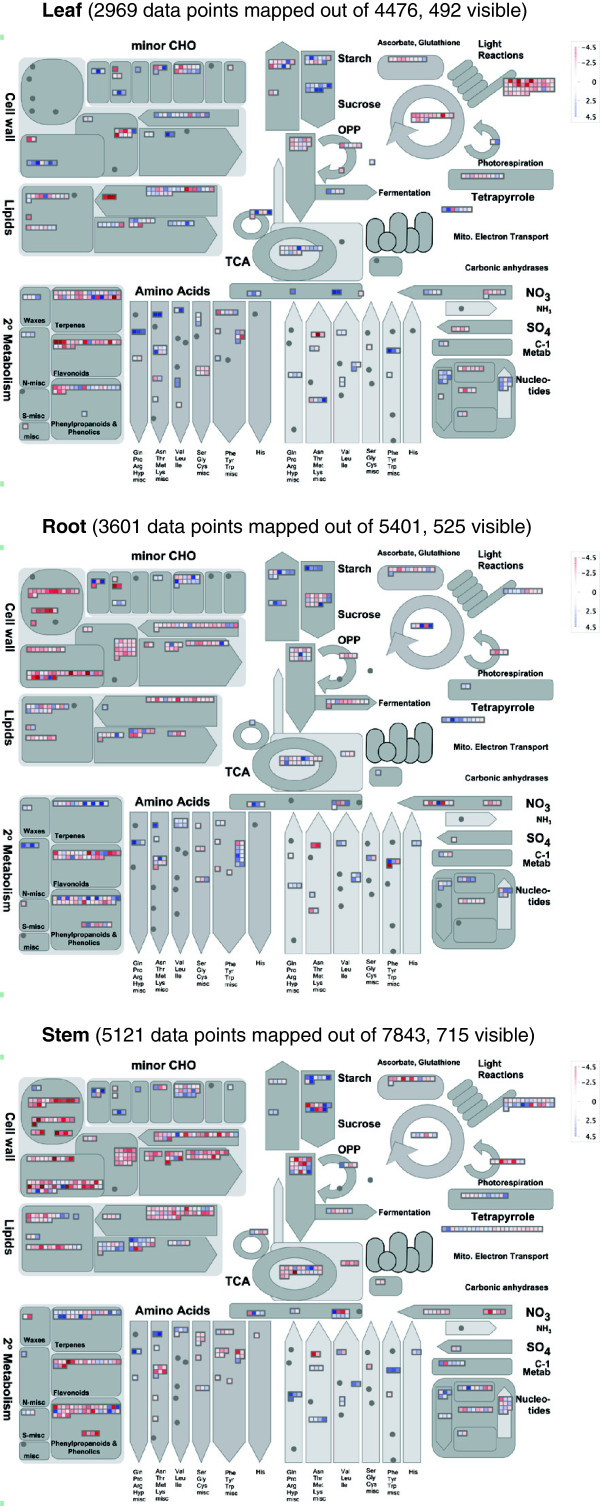
**Metabolism overview under severe water stress (optimal nitrogen).** Genes differentially regulated under severe water stress in leaf, root, and stem as visualized in Mapman (Thimm et al., 2004). In leaf, root and stem respectively, 4954, 5994, and 8767 entities were found to be differentially regulated under severe water stress (FC≥2, p-value≤0.05) and corresponded to 4476, 5401 and 7843 unique transcript identifiers imported in Mapman. Transcripts up- and down-regulated are represented with blue and red squares respectively. Values are log2-transformed fold changes.

Qualitatively, a gene ontology (GO) analysis also supported the idea that the processes involved in each organ in response to drought stress were quite unique. An overview of the metabolic processes transcriptionally affected by severe water stress was obtained using Mapman [24] and shown in Figure 3. A similar analysis under mild water stress is shown in Additional file 3. A gene enrichment analysis using AgriGO SEA (Singular Enrichment Analysis) Compare tool (Table 2) confirmed that the functional categories affected by the transcriptional changes were very distinct across tissues. Most of the GO terms significantly enriched in the lists of entities differentially regulated under mild or severe water stress compared to the optimal water control were organ-specific.

**Table 2:** Gene Ontology (GO) analysis

		**LEAF**				**ROOT**				**STEM**			
		**Mild water stress**		**Severe water stress**		**Mild water stress**		**Severe water stress**		**Mild water stress**		**Severe water stress**	
**GO Term**	**Description**	**FDR**	**Num**	**FDR**	**Num**	**FDR**	**Num**	**FDR**	**Num**	**FDR**	**Num**	**FDR**	**Num**
***Cellular Component***													
GO:0009579	thylakoid	4.80E-05	27	—	—	—	—	—	—	—	—	—	—
GO:0005576	extracellular region	—	—	—	—	2.00E-06	47	6.00E-05	81	4.30E-03	25	2.00E-03	96
***Molecular Function***													
GO:0003824	catalytic activity	4.10E-02	1042	2.00E-08	1065	3.20E-03	554	4.10E-07	1309	—	—	—	—
GO:0005215	transporter activity	4.10E-02	181	—	—	4.80E-04	112	9.30E-03	219	—	—	—	—
***Biological Process***													
GO:0015979	photosynthesis	3.00E-08	43	1.20E-03	29	—	—	—	—	—	—	—	—
GO:0008152	metabolic process	9.40E-06	1144	4.40E-04	1069	—	—	—	—	—	—	—	—
GO:0006519	cellular amino acid and derivative metabolic process	4.10E-03	85	7.90E-03	79	—	—	1.60E-02	93	—	—	—	—
GO:0006091	generation of precursor metabolites and energy	2.30E-02	64	—	—	—	—	—	—	—	—	—	—
GO:0005975	carbohydrate metabolic process	2.40E-02	139	1.20E-04	153	1.70E-02	79	1.90E-04	181	1.20E-03	56	1.50E-04	239
GO:0009628	response to abiotic stimulus	3.30E-02	137	—	—	—	—	9.20E-03	167	2.70E-02	48	3.10E-02	214
GO:0006629	lipid metabolic process	—	—	1.50E-02	90	—	—	2.40E-02	106	3.40E-02	32	6.10E-05	166
GO:0050896	response to stimulus	—	—	—	—	2.80E-05	138	1.00E-07	300	—	—	3.10E-02	333
GO:0006950	response to stress	—	—	—	—	5.30E-05	121	1.00E-07	269	3.20E-02	63	1.20E-02	302
GO:0000003	reproduction	—	—	—	—	4.20E-02	13	—	—	—	—	—	—
GO:0042592	homeostatic process	—	—	—	—	—	—	2.40E-02	177	1.60E-02	55	3.10E-02	235
GO:0065008	regulation of biological quality	—	—	—	—	—	—	2.40E-02	184	1.60E-02	56	4.50E-02	241
GO:0032501	multicellular organismal process	—	—	—	—	—	—	2.40E-02	172	4.40E-02	48	—	—
GO:0065007	biological regulation	—	—	—	—	—	—	—	—	3.40E-02	124	—	—

The analysis was performed using the Singular Enrichment Analysis (SEAcompare) on the AgriGO website (Du et al., 2010, http://bioinfo.cau.edu.cn/agriGO/). This tool allowed the identification of GO terms that were significantly enriched in the lists of entities differentially regulated between mild or severe water stress and well-watered control (FC≥2, p≤0.05). For each condition, the false discovery rate (FDR) and the number of entities (Num) are shown where the GO term enrichment was significant.

Clearly the photosynthetic machinery and its related processes (sucrose and starch metabolisms, Calvin cycle) were significantly affected by mild and severe drought in leaf. While transcripts associated with light reactions and Calvin cycle were down-regulated, those pertaining to starch and sucrose breakdown were up-regulated. These trends were accompanied by an increase in gene expression for the biosynthesis of a few amino acids, such as proline and asparagine. Overall, gene expression under drought suggested a decrease in photosynthetic assimilation to benefit remobilization of sugars and synthesis of targeted amino acids (Figure 3).

By contrast, components of the cell wall machinery were mostly affected in root and stem under severe water stress. Transcripts encoding enzymes from the pectin esterase bins, which include genes for arabinogalacatan synthesis, expansins, glucan transferases and leucin-rich repeat proteins, were highly down-regulated, converging towards the global remobilization of cell wall components (Figure 3).

It is worth noting that transcripts involved in nitrogen assimilation were also significantly affected during water stress in all three tissues, converging towards a down-regulation of nitrate reduction. The assimilation of ammonium was also affected but to slightly different extents depending on the tissue, with a decrease in glutamine and glutamate synthases transcripts in stem and root and an increase in leaf (Figure 3).

### The dynamic interaction between nitrogen and water treatments is responsible for substantial changes in the transcriptome

Our experiment was designed to study specifically the interactive effects of nitrogen and water stresses in a way that attempted to best mimic field conditions: chronic nitrogen limitation and transient drought. Under such conditions, the expression of some genes might be influenced by the nitrogen status, the water status, both, or the dynamic interaction between nitrogen and water. The latter category is of particular interest. For simplicity, only combinations of the following conditions were considered: optimal/limiting nitrogen, optimal/severe water stress (4 combinations for each organ). Only entities that had a mean fold change ≥ 2 in at least one of the comparisons in Table 3 were retained. An analysis of variance (ANOVA) was performed for each organ separately to simplify the model. P-values were corrected for multiple testing as described in the Methods section. Entities that had a p-value (nitrogen x water) ≤ 0.05 were considered significant in that their variation across the above set of conditions was explained by an interaction effect of nitrogen and water status. Results from the ANOVA were compared to the pairwise t-test results. Entities that had a p-value (nitrogen) > 0.05 and a p-value (water) > 0.05 in the t-tests and had a p-value (nitrogen x water) ≤ 0.05 were considered to be responsive to both nitrogen and water status. Results are summarized in Table 4.

**Table 3:** Pair-wise comparisons performed prior to the ANOVA

***Condition 1***	***vs***	***Condition 2***	***Fold change***
Optimal Nitrogen – Optimal Water		Optimal Nitrogen – Severe drought	FC(W)_optN_
Optimal Nitrogen – Optimal Water		Limiting Nitrogen – Optimal Water	FC(N)_optW_
Optimal Nitrogen – Severe drought		Limiting Nitrogen - Severe drought	FC(N)_sevW_
Limiting Nitrogen – Optimal Water		Limiting Nitrogen – Severe drought	FC(W)_limN_

Entities that displayed a fold change ≥ 2 in at least one of the comparisons below were used in the ANOVA.

**Table 4:** Number of differentially regulated entities per organ explained by nitrogen, water or the interactive effect of both stresses

	**Nitrogen**	**Water**	**Nitrogen x Water**
Leaf	1703 (122)	7260 (5016)	1905 (99)
Root	1575 (48)	6474 (4307)	1668 (20)
Stem	6309 (109)	9458 (1527)	6693 (188)
Total	8043	16046	8574
	17.2%	34.3%	18.3%

Numbers in brackets denote entities exclusively regulated by either nitrogen, water or the interaction. The percentages in the last row indicate the proportion of differentially regulated entities relative to the total number of entities on the chip.

In leaf, 1904 entities out of the 8042 that passed the 2-fold change cut-off were found to be significantly affected by the interaction of nitrogen and water status, 99 of which were exclusively explained by this interaction effect. In root, 1668 of the 6983 entities that passed the 2-fold change cut-off were found to be explained by the interaction and among those 20 were exclusively explained by the interaction. In stem, 6693 of the 10017 entities that passed the 2-fold change cut-off were explained by the interaction between nitrogen and water, 188 of which were responsive to the interaction effect.

Although water stress had much greater effect on gene expression than nitrogen stress, our results indicate that the interaction between nitrogen and water stresses affects regulation of a number of maize genes.

### The nitrogen x water interaction shapes the transcriptional response to a series of water treatments

**Figure 4:**
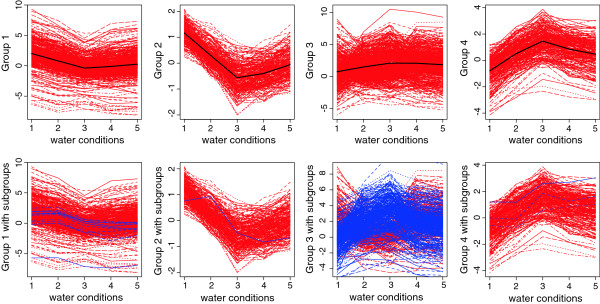
**1203 genes were selected for leaves under optimal nitrogen condition based on a fold change above 3 under either mild or severe water stress compared to optimal water.** Those genes were then classified into 4 groups shown in the graphs in the first row, where the black lines represent the respective means. The vertical axis corresponds to the normalized expression levels in the log-scale, the horizontal axis corresponds to the water treatments: optimal water supply (1), mild water stress (2), severe water stress (3), 2h after rewatering (4), 5h after rewatering (5). For each group, subgroups were arbitrarily created by splitting a posteriori (the second row). The color-coding used is blue for genes up-regulated under mild water stress (condition 2) compared to optimal water supply (condition 1) and red for genes down-regulated under the same conditions.

**Figure 5:**
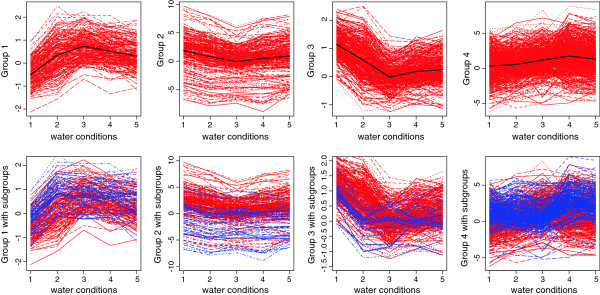
**1408 genes were selected for leaves under limiting nitrogen condition based on a fold change above 2 under either mild or severe water stress compared to optimal water.** Those genes were then classified into 4 groups shown in the graphs in the first row, where the black lines represent the respective means. The vertical axis corresponds to the normalized expression levels in log scale, the horizontal axis corresponds to the water treatments: optimal water supply (1), mild water stress (2), severe water stress (3), 2h after rewatering (4), 5h after rewatering (5). For each group, subgroups were arbitrarily created by splitting a posteriori (the second row). The color-coding used is blue for genes up-regulated under severe water stress (condition 3) compared to mild water stress (condition 2) and red for genes down-regulated under the same conditions.

The fact that we identified entities with different responses to water stress depending on the plant N status may have a profound impact on experimental designs and their results. A clustering analysis was performed to look into this issue in more detail. Only results corresponding to leaf tissue are shown here for clarity but similar trends were obtained in roots and stem unless otherwise stated. As indicated in the experimental procedure, the samples were taken simultaneously on different plants that had undergone various stress treatments. This design was driven by practical limitations and the fact that we wanted to avoid capturing the circadian component of gene expression in order to concentrate solely on stress-related responses. Nevertheless the effect of various stress conditions observed for a particular gene were not considered independent from each other. For example, the effects of the mild stress treatment may be considered intermediate to severe stress; the same goes for the 2- and 5-h re-watering time points. Hence, the data was treated as longitudinal to take into account the correlation among the measurements of gene expression over various stress conditions. A model-based clustering algorithm using a mixture of Gaussian distributions for longitudinal data, typically used to model time course gene expression studies, was used to group the genes that behaved in a similar manner over various conditions. For each entity, the median of observed values was used, as it is less susceptible to outlier than the mean. Similarly to what was performed in our previous analysis, entities were filtered based on a minimum fold-change threshold. The entities that met this criterion were then used in the model-based clustering approach. After running the data a few times, it was found that a model selecting for 1 to 8 optimal groups was adapted to our particular dataset while minimizing the computational burden. The algorithm is symmetric, resulting in both up- and down-regulated entities being grouped in the same cluster. While it makes biological sense that genes involved in similar pathways may be affected in opposite manners, subgrouping was performed a posteriori for clarity of the figures using a splitting criterion based on up- or down-regulation (Figure 4 and Figure 5, lower panels).

The first step was to identify clusters of gene expression in response to water treatment, under optimal or limiting nitrogen conditions. Under optimal nitrogen conditions, 1203 entities were found to meet a 3 fold-change cut-off for leaf and classified into four optimal gene clusters (Figure 4). A similar procedure was followed under the limiting nitrogen conditions (Figure 5). However only 417 entities met this 3 fold-change criterion. A similar scenario was found for stem and root as well, illustrating the fact that the amplitude of gene variation was smaller under limiting nitrogen compared to optimal nitrogen treatment. In order to run the algorithm with a number of entities comparable to the ones identified under optimal nitrogen, a more relaxed cut-off was set. Using a 2 fold-change threshold, 1408 entities were identified for leaf under limiting nitrogen conditions, for which the algorithm identified 4 optimal clusters of expression.

**Figure 6:**
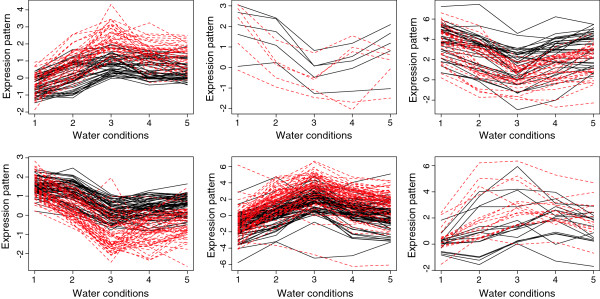
**The 228 leaves genes that showed different trends in different nitrogen conditions with black showing the gene expression under limiting nitrogen and red showing the same gene expression under optimal nitrogen.** The vertical axis corresponds to the normalized expression levels in log scale, the horizontal axis corresponds to the water treatments: optimal water supply (1), mild water stress (2), severe water stress (3), 2h after rewatering (4), 5h after rewatering (5).

The second step of the cluster analysis was to compare the genes identified as belonging to a cluster under either optimal or limiting nitrogen conditions. For leaf, 697 entities met the fold change criteria under both optimal and limiting nitrogen, and hence were assigned a cluster in both cases. The expression levels of the entities that belonged to a particular group were taken both from limiting and optimal nitrogen conditions and a model based clustering approach was run. The goal was to identify entities that had different transcriptional trends in response to water treatment depending whether nitrogen was limiting or optimal. Among the 697 entities matching the fold change threshold under both optimal and limiting nitrogen conditions for leaf, 228 entities (32.7%) were found to follow different trends under optimal and limiting nitrogen. The behaviour of those entities is shown in Figure 6. A similar analysis was run for stem and root tissue: respectively 28 out of 45 entities (62.2%) and 121 out of 277 entities (43.7%) were found to behave differently in response to the series of water treatments under limiting and optimal nitrogen supply. This analysis shows that both expression levels and patterns of expression are influenced by nitrogen and water availability. Therefore, a large proportion of entities present in one cluster under optimal nitrogen may be separated into a significantly different cluster when nitrogen is limiting.

## Discussion

In this study, we set out to examine the dynamic interaction between two of the most common abiotic stresses encountered in the field, nitrogen limitation and drought. Our experimental setup was established empirically in the greenhouse so as to achieve meaningful and controllable levels of stresses. Recovery from drought was also studied as it is a typical occurrence in the field. Under our particular experimental design, nitrogen stress was applied in a chronic and mild manner, translating into limited transcriptional changes. By contrast, drought was applied in a more severe and transient manner, leading to profound changes in transcript levels in all tissues analyzed. These changes are in agreement with the data obtained by other groups (for example, [17,21,25-27]) and with our current understanding of drought response in corn and other plant species. The main changes observed suggested a reduction of photosynthetic activity, an increase in the breakdown of complex sugars and cell wall components for remobilization, the biosynthesis of a few specific amino acids such as proline, and an adjustment of nitrogen metabolism likely in response to the decrease in carbon fixation.

Although nitrogen assimilation and therefore its metabolism are directly affected by limiting water conditions, the extent and the nature of the interaction between nitrogen stress and drought have not been looked at on a global scale to date. By focusing on the transcriptional changes occurring under limiting nitrogen conditions and water stress applied in combination, our study enabled us to establish the existence of a statistically significant interaction effect on several genes. The interaction effect was most pronounced under mild drought stress. It was surprising to find that, despite the fact that the individual effect of nitrogen limitation was not severe in this study, the N stress applied had a large impact on gene expression changes in response to water stress; especially mild water stress. This underlines the importance of a pre-existing stress such as nitrogen limitation on plants ability to respond to drought.

Our results led us to conduct a more detailed analysis of the interaction by looking at the overall pattern of gene expression during a series of drought and recovery treatments in combination with nitrogen limitation. A clustering analysis was performed that identified principal transcriptional behaviours of entities over the course of five water conditions (optimal supply, mild water stress, severe water stress, 2h recovery and 5h recovery), under optimal or limiting nitrogen supply. The defined patterns were then compared and our results indicated that almost a third of those entities identified in leaf were found to follow statistically significantly different patterns of expression in response to drought and recovery depending whether nitrogen was limiting or not. Similar trends were obtained in stem and root tissue. This finding supports the idea that nitrogen and water status were not only significant factors affecting gene expression in terms of fold change but also in terms of overall pattern of expression over a range of conditions.

From a methodological standpoint, our results demonstrate the importance of carefully controlling for various parameters when conducting expression analyses. As most studies focus mainly on a single stress and therefore often closely monitor a single parameter, the existence of additional, potentially significant secondary stresses, is often overlooked. This could explain why it can sometimes be challenging to reproduce results in different laboratories, greenhouses and growth chamber conditions.

## Conclusions

With the large amount of data available, the agricultural biosciences are now facing new challenges, which lie in our analysis and synthesis capabilities. As this study exemplifies, taking multiple factors into account is necessary to produce accurate and meaningful data. Developing tools that can integrate not only multiple environmental factors but also diverse genetic backgrounds and levels of analysis will be instrumental in finding the answers to those challenges. While such tools can already be used to predict some aspects of plant physiology for example, there is still a large gap between the impressive amount of available data and our limited understanding of biological networks and phenomena. A sustained collaborative effort between agronomists, molecular biologists, biochemists and computer scientists is the first step to provide those answers.

## Methods

### Growth conditions and physiological measurements

*Zea mays* (inbred line SRG-200) plants were grown in a semi-hydroponic system consisting of 2 L plastic pails filled with “turface”, a baked montmorillonite clay (International Minerals and Chemical, Blue Mountain, ON, Canada), and placed in trays irrigated with controlled amounts of solution. Plants were fertilized once at the beginning of the experiment with a 6 L-solution containing 187 mg/L HPO_4_ (850 g.kg^–1^), 375 mg/L KHCO_3_ (0-0-47), 400 mg/L MgSO_4_.7H_2_O (100 g.kg^–1^), 30 mg/L of Plant-Prod chelated micronutrient mix (all nutrients supplied by Plant Products, Bramalea, ON, Canada), and supplemented with ammonium nitrate (NH_4_NO_3_, 34-0-0). Concentrations of 20 mM and 8 mM NH_4_NO_3_ were defined respectively as optimal and limiting nitrogen conditions. The pH was adjusted to 5.5 to 6.0 with HCl. Plants were subsequently irrigated with purified water up to a 6 L-mark every 2 to 3 d and grown in the greenhouse under a long-day regime of 16 h light (~500 μmol m^-2^.s^-1^) at 29°C, and 8 h dark at 23°C.

At week 4, plants were treated with one of the three water treatments defined as optimal water supply, mild water stress or severe water stress. Plants grown under optimal water conditions were watered regularly up to the 6 L-mark throughout week 4. Plants grown under mild and severe stress conditions were watered regularly up to the 6 L-mark until respectively day 3 and day 5 before sampling. At that time, any residual water at the bottom of the trays was drained and the plants were not irrigated until sampling. Two re-watering treatments were performed the day of sampling by re-supplying water to severely stressed plants respectively 2 h and 5 h prior to sampling.

Stems, leaves and roots were harvested separately at the end of the fourth week. Three biological replicates were collected for each condition and frozen in liquid nitrogen. The tissue collected from the leaves consisted of the top 30 cm of the first fully expanded leaf (which corresponded to leaf 6 or 7 depending on the developmental stage of the plants given their stress treatment). The tissue collected from the stem consisted in the 15-cm region below the first node. The root system was extracted from the Turface substrate and collected.

Leaf discs of 1-inch diameter were removed using a leaf punch about 15 cm from the tip of first and second fully expanded leaves, taking care to avoid the mid-vein. Each sample was placed in a pre-weighed airtight tube and weighed to obtain the fresh weight (FW). The samples were then hydrated in distilled water with gentle shaking at 4°C overnight and weighed to obtain the turgid weight (TW). Water was then discarded and the samples were finally dried overnight in an oven and weighed again to obtain the dry weight (DW). The RWC was calculated according to the formula: RWC (%) = [(FW-DW) / (TW-DW)] x 100. The results shown are averages of four biological replicates.

### Microarray

All tissues were ground in liquid nitrogen and RNA was extracted using TRI-Reagent (Sigma-Aldrich Co. MO, USA) following the manufacturer’s instructions. Samples were DNase-treated using RQ1 RNase-free DNase (Promega Co., WI, USA). Total RNA was quantified using a Nanodrop 2000c spectrophotometer (Thermo Fisher Scientific Inc., MA, USA). Samples were further processed at Syngenta. Briefly, total RNA was reverse transcribed. Complementary RNA was synthesized *in vitro* from double stranded cDNA, biotin-labeled and hybridized to a Syngenta custom-designed maize GeneChip microarray, manufactured by Affymetrix. The GeneChip was originally designed to represent 82,000 unique maize expressed sequence tag (EST) clusters and genes as well as various negative, spike, and transgenic control genes [28]. A customized Chip Definition File (CDF) was created for this GeneChip to produce probe sets that represented gene models from the completed maize genome [29]. In addition, the custom CDF was mapped to a series of full-length cDNAs [30]ftp://ftp.ncbi.nih.gov/repository/UniGene/Zea_mays/, http://www.maizecdna.org/ as well as Maize Illumina Expressed Sequence Tags (runs corresponding to leaf, shoot apical meristem and seedlings, NCBI Sequence Read Archive http://www.ncbi.nlm.nih.gov/sra). The chip definition file contains coordinate information on 558,801 probes assigned to 46,681 probe sets. Probe sets were annotated either directly from the maize genome site (Gene Ontology and Interproscan, http://www.maizesequence.org) or using BLAST (Gene ontology and KEGG pathways) and InterProScan (Zdobnov and Apweiler, 2001). Data analysis and subsequent statistical tests were performed using Genespring GX version 11 (Agilent Technologies, CA, USA). In all analyses entities were filtered as described in the text based on their raw signal intensities. To identify genes regulated by nitrogen, entities were filtered based on a fold change above 2 between the two conditions: optimal nitrogen-optimal water and limiting nitrogen-optimal water. The statistical test performed was an unpaired t-test based on equal variance. P-values were corrected for multiple comparison using the Benjamini-Hochberg method. To identify genes regulated by mild water stress and severe water stress respectively, entities were filtered based on a fold change above 2 between the two conditions: optimal nitrogen-optimal water and optimal nitrogen-mild water stress, or optimal nitrogen-optimal water and optimal nitrogen-severe water stress. Similar statistical tests were performed as in the identification of nitrogen-regulated genes. To identify genes regulated by nitrogen, water stress, and their interaction, entities were first filtered based on a fold change above 2 in at least one of the comparisons described in Table 3. The statistical test performed was a 2-way analysis of variance (ANOVA), which uses type-III sum of square. Similarly to single factor analyses, a correction of the p-values for multiple testing was performed using the Benjamini-Hochberg method. Pathway analyses were performed in Mapman (Thimm et al., 2004, version 3.5.0 beta using *Zea mays* mapping Zm_B73_5b_FGS_cds_2011) and AgriGO (Du et al., 2010, http://bioinfo.cau.edu.cn/agriGO).

### Clustering analysis

Model-based clustering (see [31]) was utilized for this analysis. The model-based approach assumes that the population consists of a finite mixture of subpopulations and provides the flexibility to model these complex data through the imposition of a mixture of component densities. One (or more) of these component densities is then taken to represent a cluster. In this analysis, we utilized a mixture of Gaussian distributions so each subpopulation was modeled using a Gaussian distribution. The covariance structure takes into account the relationship between expression levels at different water conditions (cf. [32]). Genes are clustered into groups (or subpopulations) which have similar patterns over different water / nitrogen / water and nitrogen conditions. The cluster membership was estimated using an expectation-maximization algorithm (see [32] for details). For the analysis, expression levels of genes over different water stress levels were clustered for both nitrogen conditions and all organs separately to identify the genes with similar expression profiles. These genes could potentially have similar functions and /or may be involved in a similar biological pathway. The expression levels of the genes that met the prescreening criterion for both nitrogen conditions were clustered to check if they had the same expression profiles. Genes showing no change in their expression pattern under different nitrogen conditions should belong to the same cluster whereas genes showing different expression patterns under limiting and optimal nitrogen should belong to different clusters. With this in mind, we ran a two-component mixture model to identify genes showing different expression patterns under limiting and optimal nitrogen conditions. For illustration, if a gene X had a similar expression under both nitrogen conditions, the expression level of gene X for both limiting and optimal nitrogen belonged to group 1, say. However, if a gene Y had a change in expression pattern under limiting and optimal nitrogen, the expression pattern under optimal nitrogen was clustered in group 1 and the expression pattern under limiting nitrogen was clustered in group 2, say. Mathematical details on the algorithm can be found in [32]. The algorithm was implemented in R [33].

## Competing interests

The authors declare that they have no competing interests.

## Authors’ contributions

SH developed conditions for plant growth, performed physiological analysis, did primary analysis of microarray data, wrote the initial manuscript. SS did clustering analysis of microarray data and helped with other statistical analyses. JC did some of the microarray analysis, helped write manuscript on analysis of micorarrays. BZ helped develop conditions for maize growth conditions and helped set up these experiments. YMB helped design experiments for combined stress and helped with the microarray analysis. XC helped design experiments for combined stress. TZ supervised microarray production and helped with experimental design. PDM helped design and supervise clustering analysis, contributed to manuscript editing. SJR supervised experimental design for combined stress experiment; supervised and discussed data collected for physiological experiments and microarray analysis, contributed to manuscript design and editing. All authors read and approved the final manuscript.

## Supplementary Material

Additional file 1 **Phenotype of plants treated with different combinations of water and nitrogen stresses at time of tissue sampling.** One representative plant is shown in all cases. A. Side-by-side comparison of five week old plants grown under optimal and low nitrogen combined with optimal water treatment or mild or severe water stress, B. Close-up view of first fully expanded leaves, C. Five week old plants treated with all the stress combinations used in this study as indicated on pictures, D. Side-by-side comparison of five week old plants grown under optimal or low nitrogen (well-watered). Click here for file

Additional file 2 **Whisker plot of normalized intensity values of all chips.** The bottom and top of the boxes respectively show the lower and upper quartiles, while the middle line represents the median of the data. The whiskers indicate respectively 1.5 inter-quartile below and above the lower and upper quartiles Click here for file

Additional file 3 **Metabolism overview under mild water stress (optimal nitrogen).** Genes differentially regulated under mild water stress in leaf, root, and stem as visualized in Mapman (Thimm et al., 2004). In leaf, root and stem respectively, 5151, 2370, and 1539 entities were found to be differentially regulated under mild water stress (FC≥2, p-value≤0.05) and corresponded to 4627, 2188 and 1411 unique transcript identifiers imported in Mapman. Transcripts up- and down-regulated are represented with blue and red squares respectively. Values are log2-transformed fold changes. Click here for file

## References

[B1] Word Population Prospects The 2010 Revision United Nations: Department of Economic and Social Affairs http://www.un.org/esa/population

[B2] Backlund P Janetos A Schimel D Hatfield J Boote K Fay P Hahn L Izaurralde C Kimball BA Mader T The Effects of Climate Change on Agriculture, Land Resources, Water Resources, and Biodiversity in the United States 2008 U.S: Department of Agriculture 362

[B3] Li Y White R Chen D Zhang J Li B Zhang Y Huang Y Edis R A spatially referenced water and nitrogen management model (WNMM) for (irrigated) intensive cropping systems in the North China Plain Ecol Model 2007 203 3–4 395 423

[B4] Zand-Parsa S Sepaskhah AR Development and evaluation of integrated water and nitrogen model for maize Agricultural Water Management 2006 81 3 227 256 10.1016/j.agwat.2005.03.010

[B5] Di Paolo E Rinaldi M Yield response of corn to irrigation and nitrogen fertilization in a Mediterranean environment Field Crops Research 2008 105 3 202 210 10.1016/j.fcr.2007.10.004

[B6] Bi YM Wang RL Zhu T Rothstein SJ Global transcription profiling reveals differential responses to chronic nitrogen stress and putative nitrogen regulatory components in Arabidopsis BMC Genomics 2007 8 1 281 10.1186/1471-2164-8-281 17705847 PMC1994689

[B7] Gutiérrez RA Lejay LV Dean A Chiaromonte F Shasha DE Coruzzi GM Qualitative network models and genome-wide expression data define carbon/nitrogen-responsive molecular machines in Arabidopsis Genome Biol 2007 8 1 R7 10.1186/gb-2007-8-1-r7 17217541 PMC1839130

[B8] Gutierrez RA Stokes TL Thum K Xu X Obertello M Katari MS Tanurdzic M Dean A Nero DC McClung CR Systems approach identifies an organic nitrogen-responsive gene network that is regulated by the master clock control gene CCA1 Proc Natl Acad Sci 2008 105 12 4939 4944 10.1073/pnas.0800211105 18344319 PMC2290744

[B9] Scheible WR Morcuende R Czechowski T Fritz C Osuna D Palacios-Rojas N Schindelasch D Thimm O Udvardi MK Stitt M Genome-wide reprogramming of primary and secondary metabolism, protein synthesis, cellular growth processes, and the regulatory infrastructure of Arabidopsis in response to nitrogen Plant Physiol 2004 136 1 2483 2499 10.1104/pp.104.047019 15375205 PMC523316

[B10] Wang R Guegler K LaBrie ST Crawford NM Genomic analysis of a nutrient response in Arabidopsis reveals diverse expression patterns and novel metabolic and potential regulatory genes induced by nitrate Plant Cell 2000 12 8 1491 1509 10948265 10.1105/tpc.12.8.1491 PMC149118

[B11] Wang R Okamoto M Xing X Crawford NM Microarray analysis of the nitrate response in Arabidopsis roots and shoots reveals over 1,000 rapidly responding genes and new linkages to glucose, trehalose-6-phosphate, iron, and sulfate metabolism Plant Physiol 2003 132 2 556 567 10.1104/pp.103.021253 12805587 PMC166997

[B12] Lian X Wang S Zhang J Feng Q Zhang L Fan D Li X Yuan D Han B Zhang Q Expression profiles of 10,422 genes at early stage of low nitrogen stress in rice assayed using a cDNA microarray Plant Mol Biol 2006 60 5 617 631 10.1007/s11103-005-5441-7 16649102

[B13] Zhu G-H Zhuang C-X Wang Y-Q Jiang L-R Peng X-X Differential Expression of Rice Genes Under Different Nitrogen Forms and Their Relationship with Sulfur Metabolism Journal of Integrative Plant Biology 2006 48 10 1177 1184 10.1111/j.1744-7909.2006.00332.x

[B14] Oktem HA Eyidogan F Selcuk F Oz MT da Silva JA T Yucel M Revealing Response of Plants to Biotic and Abiotic Stresses with Microarray Technology Genes, Genomes and Genomics 2008 2 1 14 48

[B15] Kankainen M Brader G Toronen P Palva ET Holm L Identifying functional gene sets from hierarchically clustered expression data: map of abiotic stress regulated genes in Arabidopsis thaliana Nucleic Acids Res 2006 34 18 e124 10.1093/nar/gkl694 17003050 PMC1636450

[B16] Seki M Narusaka M Ishida J Nanjo T Fujita M Oono Y Kamiya A Nakajima M Enju A Sakurai T Monitoring the expression profiles of 7000 Arabidopsis genes under drought, cold and high-salinity stresses using a full-length cDNA microarray The Plant Journal 2002 31 3 279 292 10.1046/j.1365-313X.2002.01359.x 12164808

[B17] Huang D Wu W Abrams SR Cutler AJ The relationship of drought-related gene expression in Arabidopsis thaliana to hormonal and environmental factors J Exp Bot 2008 59 11 2991 3007 10.1093/jxb/ern155 18552355 PMC2504347

[B18] Degenkolbe T Do PT Zuther E Repsilber D Walther D Hincha DK Kohl KI Expression profiling of rice cultivars differing in their tolerance to long-term drought stress Plant Mol Biol 2009 69 1–2 133 153 18931976 10.1007/s11103-008-9412-7 PMC2709230

[B19] Zhou J Wang X Jiao Y Qin Y Liu X He K Chen C Ma L Wang J Xiong L Global genome expression analysis of rice in response to drought and high-salinity stresses in shoot, flag leaf, and panicle Plant Mol Biol 2007 63 5 591 608 10.1007/s11103-006-9111-1 17225073 PMC1805039

[B20] Li FH Fu FL Sha LN Li WC Identification of drought-responsive genes from maize inbred lines Zhi Wu Sheng Li Yu Fen Zi Sheng Wu Xue Xue Bao 2007 33 6 607 611 18349516

[B21] Marino R Ponnaiah M Krajewski P Frova C Gianfranceschi L Pe ME Sari-Gorla M Addressing drought tolerance in maize by transcriptional profiling and mapping Molecular Genetics & Genomics 2009 281 2 163 179 10.1007/s00438-008-0401-y 19018570

[B22] Rizhsky L Liang H Mittler R The combined effect of drought stress and heat shock on gene expression in tobacco Plant Physiol 2002 130 3 1143 1151 10.1104/pp.006858 12427981 PMC166635

[B23] Rizhsky L Liang H Shuman J Shulaev V Davletova S Mittler R When defense pathways collide The response of Arabidopsis to a combination of drought and heat stress. Plant Physiology 2004 134 4 1683 1696 10.1104/pp.103.033431 PMC419842 15047901

[B24] Thimm O Blasing O Gibon Y Nagel A Meyer S Kruger P Selbig J Muller LA Rhee SY Stitt M MAPMAN: a user-driven tool to display genomics data sets onto diagrams of metabolic pathways and other biological processes Plant J 2004 37 6 914 939 10.1111/j.1365-313X.2004.02016.x 14996223

[B25] Denby K Gehring C Engineering drought and salinity tolerance in plants: lessons from genome-wide expression profiling in Arabidopsis Trends Biotechnol 2005 23 11 547 552 10.1016/j.tibtech.2005.09.001 16165235

[B26] Lenka SK Katiyar A Chinnusamy V Bansal KC Comparative analysis of drought-responsive transcriptome in Indica rice genotypes with contrasting drought tolerance Plant Biotechnology Journal 2011 9 3 315 327 10.1111/j.1467-7652.2010.00560.x 20809928

[B27] Wang D Pan Y Zhao X Zhu L Fu B Li Z Genome-wide temporal-spatial gene expression profiling of drought responsiveness in rice BMC Genomics 2011 12 149 10.1186/1471-2164-12-149 21406116 PMC3070656

[B28] Coneva V Zhu T Colasanti J Expression differences between normal and indeterminate1 maize suggest downstream targets of ID1, a floral transition regulator in maize J Exp Bot 2007 58 13 3679 3693 10.1093/jxb/erm217 17928372

[B29] Schnable PS Ware D Fulton RS Stein JC Wei F Pasternak S Liang C Zhang J Fulton L Graves TA The B73 maize genome: complexity, diversity, and dynamics Science 2009 326 5956 1112 1115 10.1126/science.1178534 19965430

[B30] Soderlund C Descour A Kudrna D Bomhoff M Boyd L Currie J Angelova A Collura K Wissotski M Ashley E Sequencing, mapping, and analysis of 27,455 maize full-length cDNAs PLoS Genet 2009 5 11 e1000740 10.1371/journal.pgen.1000740 19936069 PMC2774520

[B31] Fraley C Raftery AE Model-Based Clustering, Discriminant Analysis, and Density Estimation J Am Stat Assoc 2002 97 458 611 631 10.1198/016214502760047131

[B32] McNicholas PD Murphy TB Model-based clustering of longitudinal data Can J Stat 2010 38 1 153 168

[B33] Team RDC R: A Language and Environment for Statistical Computing R Foundation for Statistical Computing 2011 Austria: Vienna

