# Supplementary material for: Genome-wide expression profiling of maize in response to individual and combined water and nitrogen stresses
Source: BMC Genomics. 2013 Jan 16;14:3. doi: 10.1186/1471-2164-14-3 (PMC3571967; doi:10.1186/1471-2164-14-3)

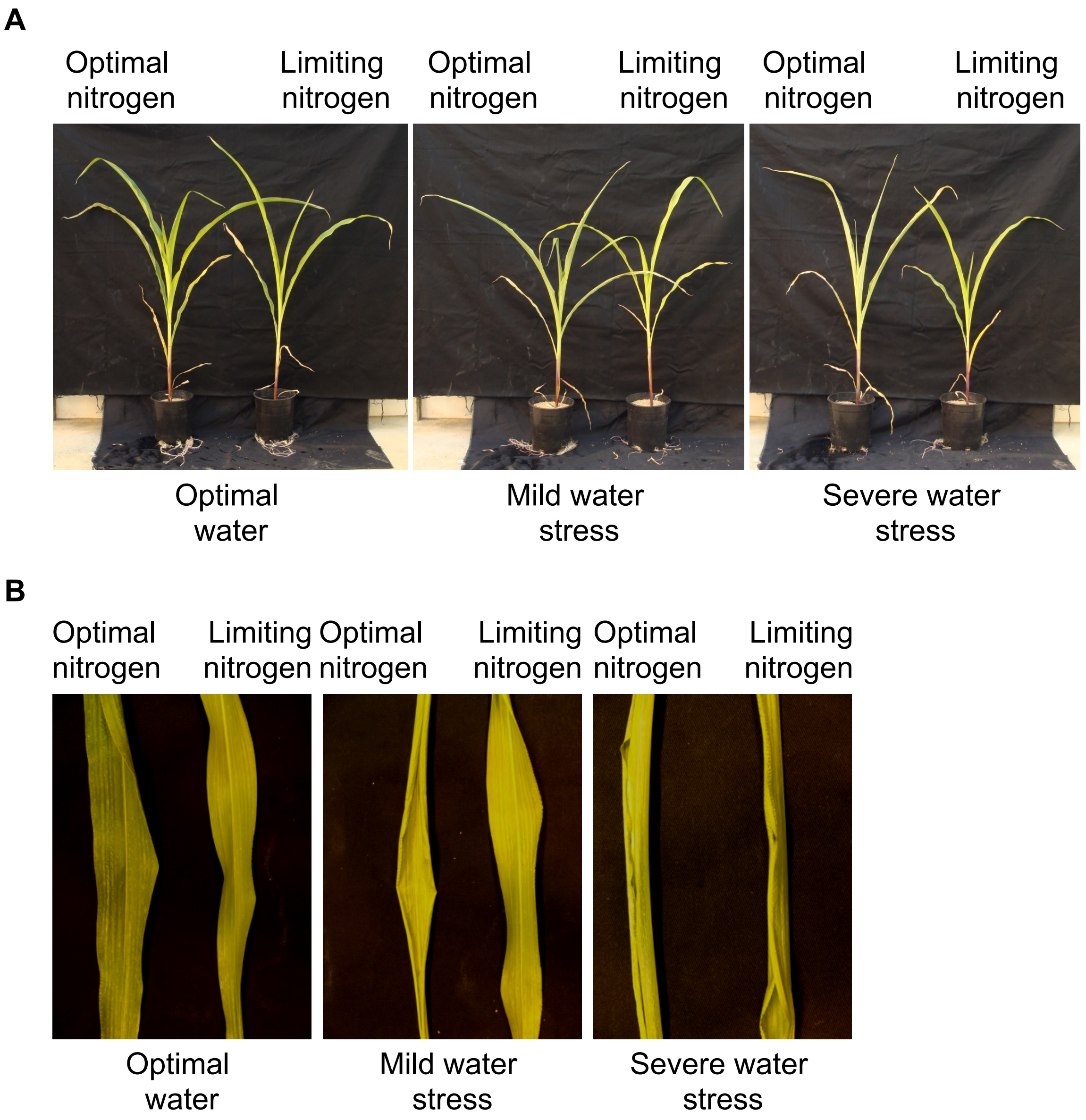

A
Optimal nitrogen
Limiting nitrogen
Optimal nitrogen
Limiting nitrogen
Optimal nitrogen
Limiting nitrogen
Optimal water
Mild water stress
Severe water stress
B
Optimal nitrogen
Limiting nitrogen
Optimal nitrogen
Limiting nitrogen
Optimal nitrogen
Limiting nitrogen
Optimal water
Mild water stress
Severe water stress

C

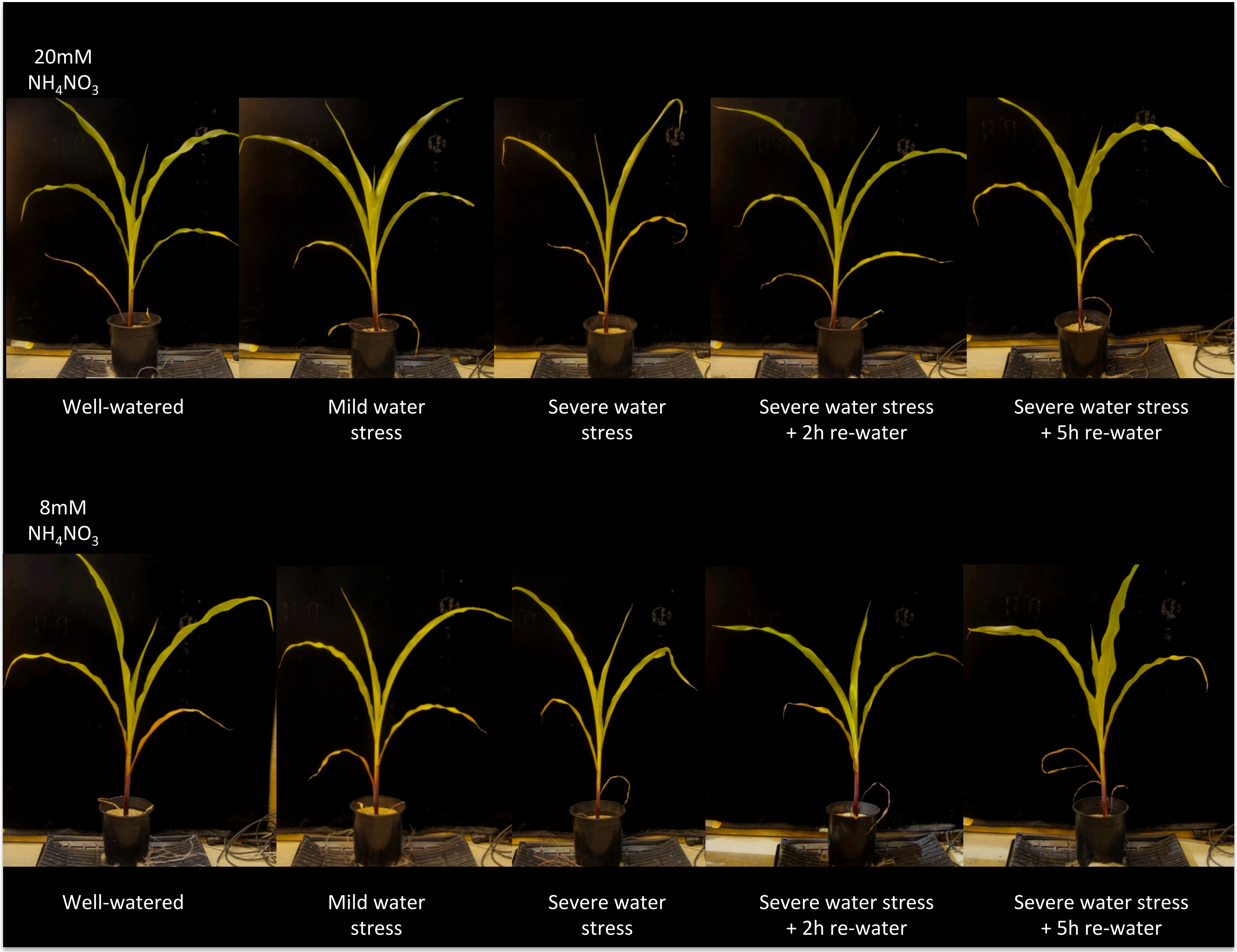
20mM
$NH_4NO_3$
Well-watered
Mild water
stress
Severe water
stress
Severe water stress
+ 2h re-water
Severe water stress
+ 5h re-water
8mM
$NH_4NO_3$
Well-watered
Mild water
stress
Severe water
stress
Severe water stress
+ 2h re-water
Severe water stress
+ 5h re-water

**D**

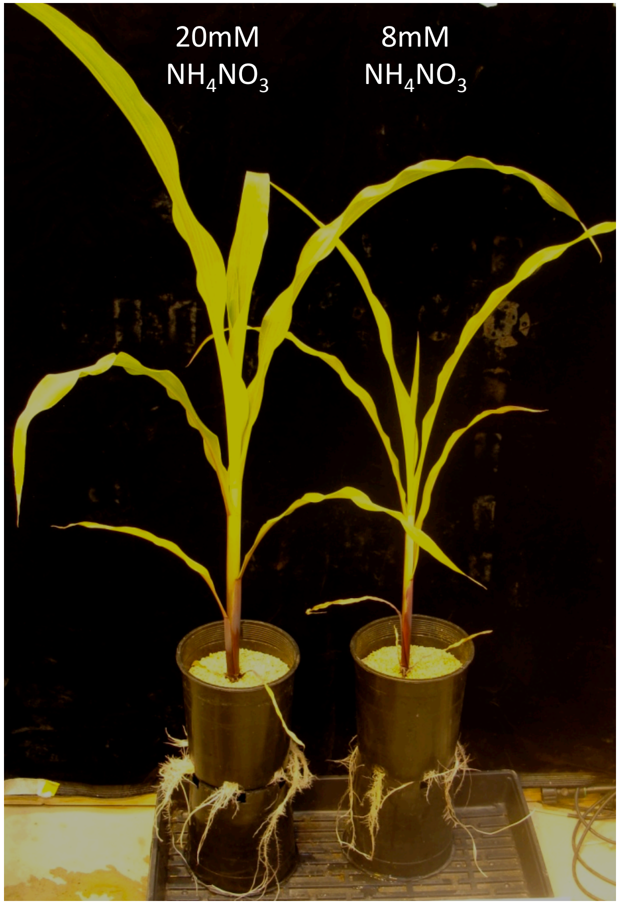

Supplement: Additional file 1 — Phenotype of plants treated with different combinations of water and nitrogen stresses at time of tissue sampling. One representative plant is shown in all cases. A. Side-by-side comparison of five week old plants grown under optimal and low nitrogen combined with optimal water treatment or mild or severe water stress, B. Close-up view of first fully expanded leaves, C. Five week old plants treated with all the stress combinations used in this study as indicated on pictures, D. Side-by-side comparison of five week old plants grown under optimal or low nitrogen (well-watered). [file 1471-2164-14-3-S1.pdf]
