# Supplementary figures and images for: Genome-wide expression profiling of maize in response to individual and combined water and nitrogen stresses
Source: BMC Genomics. 2013 Jan 16;14:3. doi: 10.1186/1471-2164-14-3 (PMC3571967; doi:10.1186/1471-2164-14-3)

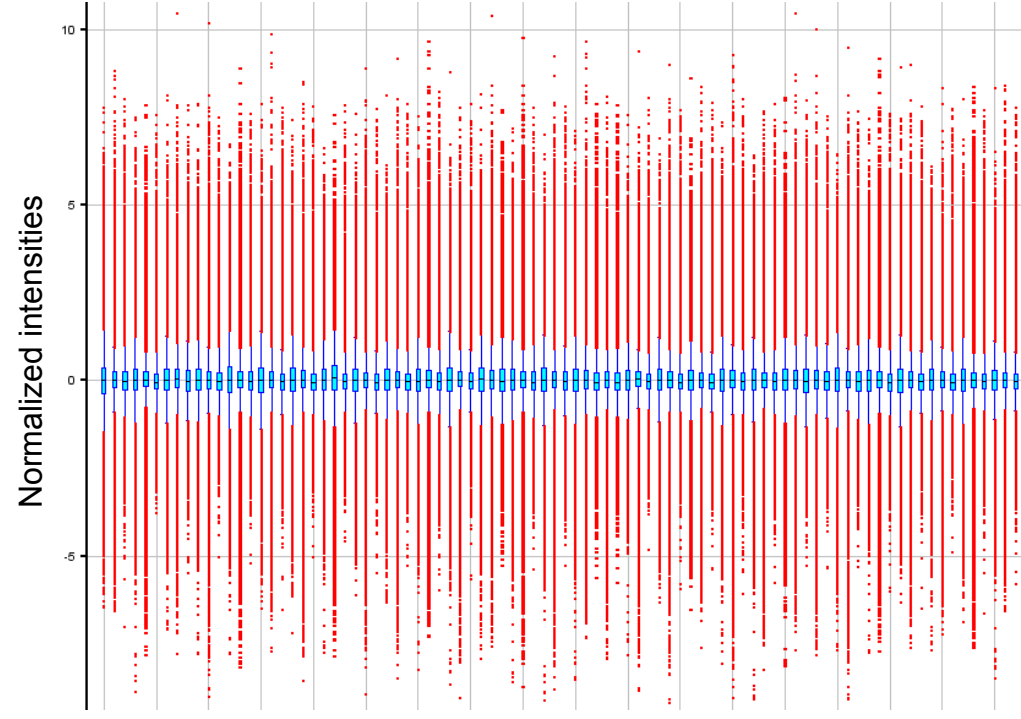

Supplement: Additional file 2 — Whisker plot of normalized intensity values of all chips. The bottom and top of the boxes respectively show the lower and upper quartiles, while the middle line represents the median of the data. The whiskers indicate respectively 1.5 inter-quartile below and above the lower and upper quartiles [file 1471-2164-14-3-S2.pdf]
