# Supplementary material for: Genome-wide expression profiling of maize in response to individual and combined water and nitrogen stresses
Source: BMC Genomics. 2013 Jan 16;14:3. doi: 10.1186/1471-2164-14-3 (PMC3571967; doi:10.1186/1471-2164-14-3)

Leaf (3039 data points mapped out of 4627, 513 visible)

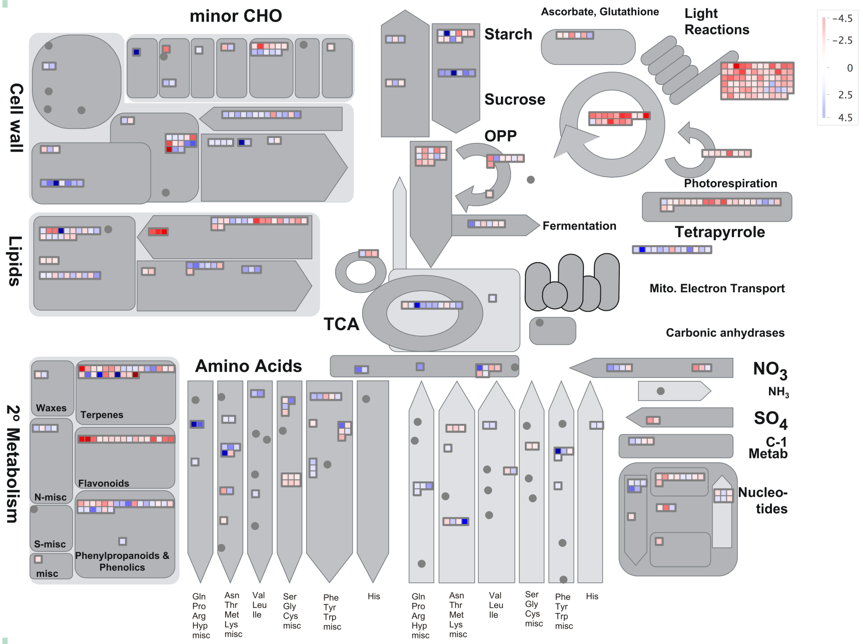

Root (1546 data points mapped out of 2188, 210 visible)

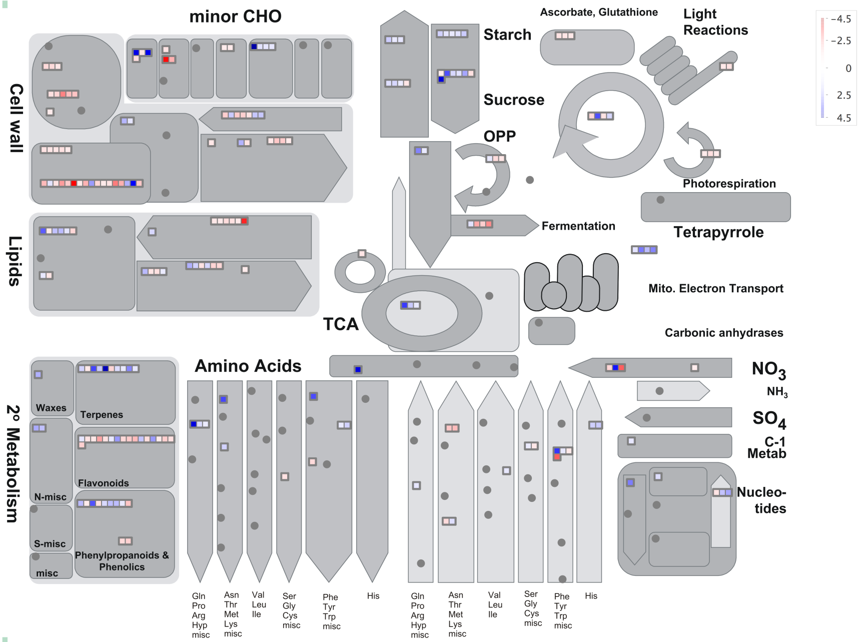

Stem (927 data points mapped out of 1411, 153 visible)

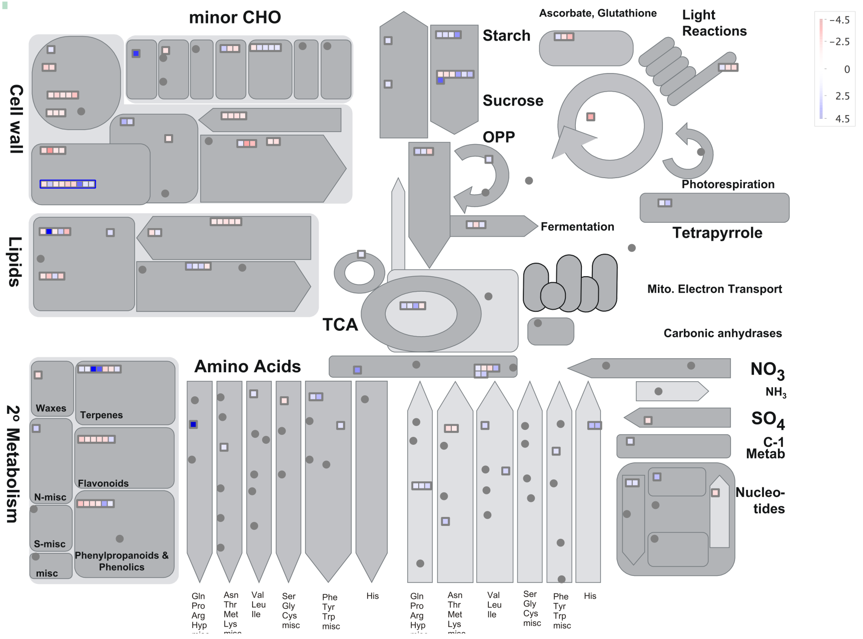

Supplement: Additional file 3 — Metabolism overview under mild water stress (optimal nitrogen). Genes differentially regulated under mild water stress in leaf, root, and stem as visualized in Mapman (Thimm et al., 2004). In leaf, root and stem respectively, 5151, 2370, and 1539 entities were found to be differentially regulated under mild water stress (FC≥2, p-value≤0.05) and corresponded to 4627, 2188 and 1411 unique transcript identifiers imported in Mapman. Transcripts up- and down-regulated are represented with blue and red squares respectively. Values are log2-transformed fold changes. [file 1471-2164-14-3-S3.pdf]
